# Predicting airway immune responses and protection from immune parameters in blood following immunization in a pig influenza model

**DOI:** 10.3389/fimmu.2024.1506224

**Published:** 2024-12-19

**Authors:** Simon Gubbins, Basudev Paudyal, Barbara Dema, Ashutosh Vats, Marta Ulaszewska, Eleni Vatzia, Elma Tchilian, Sarah C. Gilbert

**Affiliations:** ^1^ The Pirbright Institute, Pirbright, United Kingdom; ^2^ Nuffield Department of Medicine, Pandemic Sciences Institute, University of Oxford, Oxford, United Kingdom; ^3^ Chinese Academy of Medical Science (CAMS), Oxford Institute (COI), University of Oxford, Oxford, United Kingdom

**Keywords:** influenza A virus, pig, mucosal immunity, bronchoalveolar lavage, aerosol, protection

## Abstract

Whereas the intranasally delivered influenza vaccines used in children affect transmission of influenza virus in the community as well as reducing illness, inactivated influenza vaccines administered by intramuscular injection do not prevent transmission and have a variable, sometimes low rate of vaccine effectiveness. Although mucosally administered vaccines have the potential to induce more protective immune response at the site of viral infection, quantitating such immune responses in large scale clinical trials and developing correlates of protection is challenging. Here we show that by using mathematical models immune responses measured in the blood after delivery of vaccine to the lungs by aerosol can predict immune responses in the respiratory tract in pigs. Additionally, these models can predict protection from influenza virus challenge despite lower levels of blood responses following aerosol immunization. However, the inclusion of immune responses measured in nasal swab eluates did not improve the predictive power of the model. Our models are an important first step, providing proof of principle that it is feasible to predict immune responses and protection in pigs. This approach now provides a path to develop correlates of protection for mucosally delivered vaccines in samples that are easily accessed in clinical trials.

## 1 Introduction

Immunization against infectious diseases is the most cost-effective public health measure. Extensive vaccination campaigns resulted in the eradication of smallpox in humans and rinderpest in livestock. Although not yet complete, polio eradication may also be achieved (1) and the widely used vaccine against measles is sufficiently effective for that disease to also be a target for eradication (2). However, for common respiratory infections such as influenza, respiratory syncytial virus and SARS-CoV-2, the aim of vaccination campaigns is not to eradicate the disease, prevent transmission or even to prevent infection, but to prevent illness, which is severe enough to require medical attention, and to prevent deaths, even when revaccination occurs frequently. For any pathogen with a zoonotic reservoir such as influenza A, eradication can never be achieved as spillover into humans may recur. However, such events are rare, and a vaccine capable of preventing transmission of influenza A between humans could ultimately be used to reduce infections to occasional small outbreaks which may be contained locally, as is now the case with Ebolavirus. Influenza B, which has no animal reservoir, could be a target for eradication, and the B/Yamagata lineage may already have become extinct during the Covid pandemic as a result of infection control measures which prevented human to human transmission of more than just SARS-CoV-2 (3). The annual costs of influenza-related healthcare in the US, England and Germany have been estimated as US $250-725 million, £50-64 million and €55 million, respectively (4–6). Increasing the efficacy of vaccines against respiratory infections to prevent infection and transmission is therefore warranted.

In response to influenza virus in the respiratory tract, a highly co-ordinated, compartmentalised series of innate and then adaptive responses act at the site of infection (7–9). However, vaccines, with the exception of a live attenuated influenza vaccine (LAIV) used in children, are given by intramuscular injection and therefore do not result in an equivalent response (10–13). Using the pig influenza pre-exposure model we demonstrated that immune responses to the same viral vectored vaccines (chimpanzee adenovirus and attenuated modified vaccinia Ankara virus vaccines expressing nucleoprotein, matrix protein and neuraminidase, ChAdOx-NPM1-NA2 and MVA-NPM1-NA2) administered by different routes resulted in different immune responses in the blood and respiratory tract (14, 15). Unsurprisingly immunization by intramuscular injection resulted in higher responses in the blood, and after aerosol administration to the lung, higher responses were measured in bronchoalveolar lavage fluid and nasal swabs (14, 15). Following infectious influenza A virus challenge, viral shedding was reduced, and lung viral load and pathology abrogated in both groups (15).

Pigs are an important natural host for influenza, are a source of pandemic viruses, and are an excellent model for human influenza (16–18). Pigs exhibit similar clinical manifestations and pathogenesis when infected with influenza viruses making them an excellent model to study immunity to influenza (19) This similarity extends to the lobar and bronchial anatomy, as well as the histological structure of pig lungs which closely resembles that in humans (20). Using the pig model, it is possible to obtain samples of tissue and fluids from the respiratory tract either after immunization or virus challenge. In humans, although bronchoalveolar lavage (BAL) may be performed in small studies involving healthy young adults, for the majority of clinical trials sampling is limited to blood, and swabs or washes from the upper respiratory tract only. Since responses in the blood are lower, or even undetectable, after mucosal rather than intramuscular vaccination and mucosal tissues cannot be accessed, correlates of protection have not been defined, and the more widespread use of mucosal vaccination is hampered by the fact that vaccine efficacy must be demonstrated in large, slow and expensive clinical trials for each novel vaccine or modification to a licensed vaccine. We therefore sought to use data from previously published data using the pig model to predict mucosal immune responses and protective efficacy after aerosolised ChAdOx-NPM1-NA2/MVA-NPM1-NA2 vaccine delivery to the lung. Here we show that immune parameters measured in blood samples taken at particular times after immunization can be used to predict immune response in the airways, or more robustly to define an immune response that protects against infectious virus challenge in the respiratory tract. This now provides a route into clinical development of vaccines delivered to the lung, in which blood samples may be used to predict mucosal immunity and vaccine efficacy.

## 2 Methods

### 2.1 Experimental design

In the present study, we utilized data from two previous animal experiments (15).

#### 2.1.1 Prime-boost immunization experiment

The first study involved 20 six-week old female pigs which were pre-exposed to A/swine/England/1353/2009 (referred to as pH1N1) (**Supplementary Figure S1A**). Four weeks after the pH1N1 inoculation, the pigs were randomly divided into four groups of five animals and were immunized with ChAdOx2-NPM1-NA2 intramuscularly (IM), intranasally (IN) or by aerosol (AE). The production of ChAdOx2 NPM1, ChAdOx2-NPM1-NA2, MVA-NPM1 and MVA-NPM1-NA2 vaccines has been described previously (15). The NP and M1 protein ORFs were derived from A/swine/England/1353/2009 (GenBank accession number KR701098 and KR701100) and the neuraminidase (NA2) is from H3N2 strain A/swine/Ohio/A01354299/2017 (GenBank accession number MF801571).

Four weeks after the ChAdOx2-NPM1-NA2 immunization the pigs were boosted by the same delivery route with MVA-NPM1-NA2. Unimmunized, but pH1N1 pre-exposed pigs were used as controls (C). The animals were culled four weeks after the boost, and immune responses were evaluated in the bronchoalveolar lavage (BAL), spleen and blood. Serum IgG and IgA antibody responses against pH1N1and H3N2, and IgG N2 were measured by ELISA weekly at days (D) D35, D42, D49, D56, D63, D70, D77 and D83. IFNg ELISpot responses were measured in peripheral blood mononuclear cells (PBMC) following stimulation with pools of overlapping peptides covering the NP, M1 and NA proteins included in the vaccine or following stimulation with live pH1N1 and H3N2 viruses (15). BAL IgG and IgA antibody responses against pH1N1 and H3N2 and NP-, M1-, NA-, pH1N1- and H3N2- specific IFNg ELISpot responses were enumerated at postmortem at D83 (**Supplementary Figure S1A**). In addition to the published immunological data, we also included results from Enzyme-Linked Lectin Neuraminidase Inhibitory Antibodies Assay (ELLA) performed as previously described by (21) using recombinant NA2 with tetrabrachion folder (Native Antigen Company) (**Supplementary Figure S2A**). For the modelling of BAL parameters from blood, we used immunological data from AE, IM and C animals.

#### 2.1.2 H3N2 challenge experiment

In the second experiment the efficacy of prime-boost immunization against H3N2 challenge was evaluated. Twenty-four six-week old female animals were inoculated with pH1N1. Four weeks later the pigs were randomly divided into four groups of six animals with comparable weight and immunized either IM, IN or by AE with ChAdOx2-NPM1-NA2 and MVA-NPM1-NA2 four weeks apart as described above (**Supplementary Figure S1B**). Four weeks after the MVA-NPM1-NA2 boost, all animals were infected intranasally with A/swine/Ohio/A01354299/2017 (H3N2). Animals were humanely euthanized four days later. Three animals reached their humane end point before the completion of the study due to bacterial infection unrelated to the procedures. Thus, the IN and C groups contained only four and five animals, respectively (15).

Viral load was measured by plaque assays in daily nasal swabs following H3N2 challenge and in lung, and BAL at postmortem D87. Gross and histopathological analyses were performed as previously described (22). Briefly, the lungs were removed, and digital photographs were taken of both the dorsal and ventral aspects. Utilizing image analysis software (Nikon NIS-Ar) on these images the percentage of the lung gross lesions in each animal’s lung was calculated. Macroscopic pathology was scored blindly in line with established protocols (23). Subsequently tissue samples were obtained from cranial, middle, and caudal lung lobes into 10% neutral-buffered formalin for standard histological processing with Haematoxylin and eosin staining. Immunohistochemical staining for influenza A virus nucleoprotein (NP) was performed on 4-mm tissue sections. Histopathological assessment on lung tissue sections was performed by a veterinary pathologist blinded to the treatment group. A scoring system comprising five parameters (necrosis of the bronchiolar epithelium, airway inflammation, perivascular/bronchiolar cuffing, alveolar exudates, and septal inflammation) was employed, each rated on a scale of 0–4. These scores were then summed to give a total slide score ranging from 0–20 per lobe and a cumulative animal score ranging from 0 to 60 (24). For each animal a mean score for the three lung lobes was calculated. The individual lung lobes were also scored using the “Iowa” method, which considers the amount of viral NP antigen present in the sample, as previously described.

Serum pH1N1, H3N2 and N2 IgG titers were determined by ELISA in weekly bleeds on D35, D42, D49, D56, D63, D70, D77 and at postmortem D87. BAL pH1N1 and H3N2 IgG and IgA titers were assayed at postmortem D87. NP-, M1-, NA2-, pH1N1- and H3N2- specific IFNg ELISpot responses were enumerated in PBMC and BAL at PM D87. In addition to the published immunological assays (15) we also evaluated pH1N1 and H3N2 specific IgG and IgA titers in nasal swabs by ELISA (**Supplementary Figure S2B**). Interestingly, only AE and IN immunization induced significant H3N2 specific IgA responses in nasal swabs, while IM immunization elicited the strongest IgG H3N2 specific response most likely due to antibody transudation. Data from all immunological assays is provided in **Supplementary Tables S2** and **S3**.

### 2.2 Statistical methods

**Figure 1:**
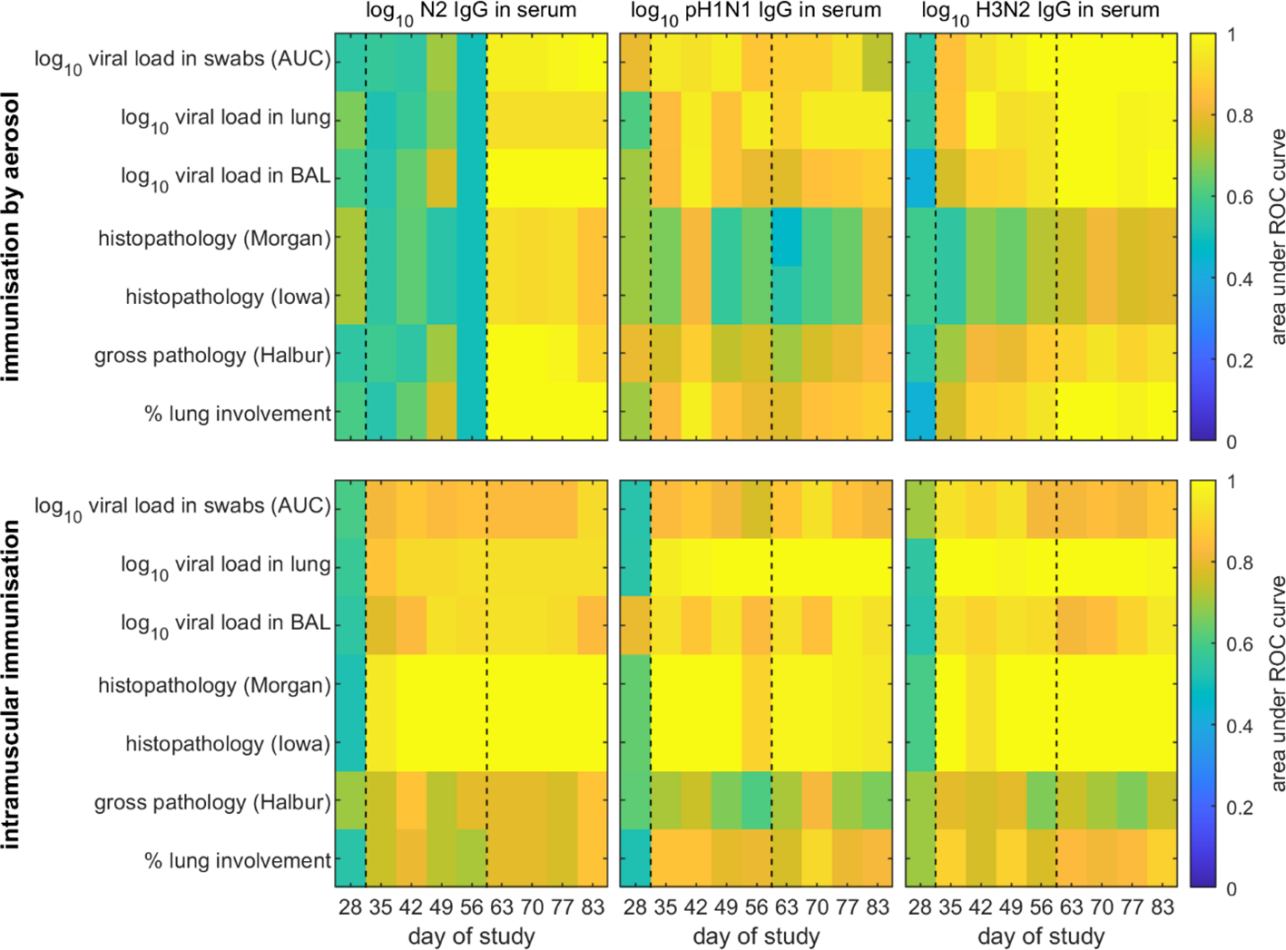
Receiver operator characteristic (ROC) analysis of models for the probability of protection in pigs following challenge with H3N2 swine influenza virus. Pigs were immunised by aerosol (top) or intramuscularly (bottom). Protection was defined as absence of virus or pathology based on different measures (listed on the *y* axis), while the probability of protection was estimated using each immune parameter (indicated above the panel) measured at a single time point (listed on the *x* axis) as a predictor. The black dashed lines indicate the timing of prime and boost vaccinations.

Models were developed to predict: (i) immune parameters in BAL from those measured in blood; and (ii) protection after challenge (i.e. presence/absence of virus or pathology) from immune parameters measured prior to challenge. The immune parameters in BAL and their predictors are listed in **Table 1** and **Supplementary Table S1** respectively. The viral loads and pathology scores and their predictors are listed in **Figure 1** and **Supplementary Figure S4** respectively. The models were implemented in Matlab (version R2020b; The Mathworks Inc.). Data for the IN immunised pigs were not included in any of the statistical analyses as none was protected following challenge (15).

**Table 1:** Models predicting immune parameters against influenza virus in broncho-alveolar lavage (BAL) from those in blood in pigs immunised by aerosol or intramuscularly.

Immune parameter in blood	Immune parameter in Broncho-Alveolar Lavage (BAL)										
	H3N2 IgG	H3N2 IgA	pH1N1 IgG	pH1N1 IgA	N2 IgG	pH1N1 ELISpot	NP ELISpot	M1 ELISpot	NA ELISpot	H3N2 ELISpot	NA2 ELLA
intercept	3.16	-3.75	-10.31	4.38	-16.40	-1.49	4.43	5.87	-1.10	13.90	0.62
H3N2 IgG serum D21					-6.42						
H3N2 IgG serum D28					8.70						
H3N2 IgG serum D56	0.90										
H3N2 IgG serum D69											-0.15
H3N2 IgA serum D28							0.6			1.50	
pH1N1 IgG serum D28		1.27	4.05	-0.05	2.49			-0.61		-2.11	-0.07
pH1N1 IgG serum D76						1.01					
pH1N1 IgA serum D69				-0.15							
pH1N1 IgA serum D83						0.68					
N2 IgG serum D42		-0.72									
N2 IgG serum D62				-0.41							
N2 IgG serum D69							-0.84				
pH1N1 ELISpot D63				0.59							
NP ELISpot D28			-2.03								
NP ELISpot D35		0.62									
NP ELISpot D42								-0.83			
NP ELISpot D56									1.34	-1.22	
M1 ELISpot D56								0.42			
NA ELISpot D28					1.68					-2.72	
NA ELISpot D42									-0.25		
NA ELISpot D63											0.05
NA ELISpot D77								-0.59			-0.16
H3N2 ELISpot D28			0.28								
H3N2 ELISpot D35									-1.12		
H3N2 ELISpot D56		0.23			-0.63						
H3N2 ELISpot D77							0.36				
NA2 ELLA D48										-1.32	
NA2 ELLA D69	-1.28										

(Values are the estimated regression coefficients; all differ significantly (*P*<0.05) from zero. The predicted value for an immune parameter in BAL (column) can be computed from the intercept plus each of the coefficients in grey in that column multiplied by the value measured for the corresponding immune parameter in blood).

#### 2.2.1 Immune parameters in BAL

Separate models were developed for each of eleven immune parameters in BAL (**Table 1**) using data for AE and IM immunised pigs. Models were constructed by stepwise linear regression of the log_10_ transformed data (both responses and predictors). Starting from an intercept only model, each predictor (**Supplementary Table S1**) was added to the model one at a time and the predictor which yielded the largest change in adjusted *r* ^2^ (above a threshold of 0.02) was added to the model. This was repeated until adding terms did not increase the adjusted *r* ^2^ by more than the threshold. Next, terms in the model were deleted from the model one at a time and were removed if the change in adjusted *r* ^2^ was less than 0.01. This was repeated until deleting terms did not decrease the adjusted *r* ^2^ by less than the threshold. The adjusted *r* ^2^ value was used as the criterion for model selection because the aim of the models is predictive rather than inferential.

The accuracy of the model for each immune parameter in BAL for out-of-sample prediction was assessed using leave-one-out cross validation. In this case, a model was fitted to the data for all pigs except one using the process described above, and the immune parameter for the omitted animal was estimated using the model and compared to the observed value.

Two additional models were considered for each immune parameter in BAL. The first additional model was constructed using only data for the same parameter measured in blood. The second additional model was constructed using only immune parameters measured at the same time point as predictors. Models were constructed using the same stepwise process described above. Where immune parameters were measured one day apart (**Supplementary Table S1**), these were assumed to be measured on the same day for the purposes of model construction.

#### 2.2.2 Protection following challenge

A pig was considered protected if it had no measurable viral load (i.e. the level of virus in nasal swabs, lung or BAL was equal to zero) or no measurable pathology (as measured by one of four pathology scores) (see **Figure 1**).

The probability (*p*) that a pig with a given level of IgG in serum or nasal swabs, *T*, was protected after challenge was given by log(*p*/(1-*p*))=*a*×(log_10_ *T*-*b*), where *a* is the slope and *b* is the level at which 50% of pigs would be protected. Parameters were estimated using Bayesian methods, assuming a Bernoulli likelihood for the data and diffuse normal priors (with mean 0 and standard deviation 10) for the parameters (*a* and *b*). Two chains each of 120,000 samples were run, with the first 20,000 iterations discarded to allow for burn-in of the chain. Chains were subsequently thinned by selecting every tenth iteration to reduce autocorrelation amongst the samples. Convergence of the chains was monitored visually and using the Gelman-Rubin statistic implemented in the coda package (25) in R (version 4.4.0) (26).

Separate models were constructed for pigs immunised by aerosol or intramuscularly for each of the seven measures of protection using only one immune parameter (log_10_ N2 IgG, log_10_ pH1N1 IgG or log_10_ H3N2 IgG in serum, log_10_ pH1N1 or H3N2 IgA or IgG in nasal swabs) measured at a single time point as a predictor (see **Figure 1**; **Supplementary Figure S5**, respectively). Model accuracy was assessed using receiver-operating characteristic (ROC) analysis (27). Specifically, the area under the ROC curve was calculated for each model (i.e. measure of protection and time point).

## 3 Results

We have previously demonstrated that intramuscular or aerosol prime boost immunization with ChAdOx2-NPM1-NA2 and MVA-NPM1-NA2 in pH1N1 pre-exposed pigs reduced viral shedding and abolished lung viral load and pathology after H3N2 challenge (15). We performed detailed analyses of antibody and T cell responses following immunization and challenge in blood, bronchoalveolar lavage (BAL), lung, tracheobronchial lymph nodes and spleen (15) (**Supplementary Figure S1**). In this study we have also included new data on immune responses: neuraminidase inhibition antibody titres in blood and antibody ELISA titres in nasal swabs (**Supplementary Figure S2**). Utilizing the data from these immunological analyses here we wish to determine, first, whether immune responses in the BAL can be predicted by immune parameters in the blood, and second, whether protection against challenge can be predicted by immune parameters in BAL, in blood or in nasal swabs.

### 3.1 Predicting immune parameters in BAL from those in blood

Separate models were constructed for eleven immune parameters in BAL, including H3N2, pH1N1, N2 specific IgG and IgA titers, NA2 ELLA and IFNg ELISpot responses against NP, M1 and H3N2 (**Table 1**) using data for pigs immunised by aerosol (AE) or intramuscularly (IM). Ninety-nine potential predictors were considered in the models, specifically serum pH1N1, H3N2 and N2 IgG and IgA ELISA titers and N1, M1, NA2 and H3N2 IFNg ELISpot responses measured in blood at different time points post immunization (**Supplementary Figure S1A**; **Supplementary Table S1**). Models were constructed by stepwise linear regression in which predictors were added to or removed from the model one at a time until adding or removing terms did not change the adjusted *r* ^2^ by more than a specified threshold.

All models were able to capture the data accurately (adjusted *r* ^2^ >0.98 for all models) (**Supplementary Figure S3**). The predictors in the final models were varied, including both antibody and T cell responses, and 30 (out of 99) predictors were included in at least one model (**Table 1**). Furthermore, predictors came from multiple time points across the experiments, both before and after prime or boost immunization (**Table 1**). For example, H3N2 IgG in the BAL can be predicted by measuring serum H3N2 IgG in serum at day 56 (D56) and NA2 ELLA in serum at D69, while H3N2 IgA in the BAL can be predicted by measuring pH1N1 IgG in serum at D28, N2 IgG in serum at D42, NP IFNγ ELISpot at D35 and H3N2 IFNγ ELISpot at D56.

To assess the ability of the models to predict responses using new data, we performed leave-one-out cross validation in which models were fitted to data for all but one pig and then used to predict the immune responses in BAL for the pig which had been omitted. Comparing the predicted responses with those observed for each pig suggests that the models are not particularly accurate when predicting responses using new data (**Supplementary Figure S4**). This was the case for all eleven immune parameters in BAL and reflects the fact that only data for a small number of pigs were used to fit the models.

Two additional models were constructed for each immune parameter in BAL, also by stepwise linear regression. In the first additional model, only the same immune parameter measured in blood at different time points were used as predictors. In the second additional model, only immune parameters measured at the same time point were used as predictors. However, model fit was generally poor (adjusted *r* ^2^<0.7) for the first additional model, while for the second additional model there was no pattern to which time points (e.g. all post booster vaccination) yielded the best-fitting model across the immune parameters in BAL. Accordingly, models for immune parameters in BAL based on a single parameter or on immune parameters in blood measured at a single time point were not considered further.

Overall, these results show that immune responses in BAL can be predicted from immune parameters in blood, although it requires multiple parameters measured at multiple time points to do so. However, the accuracy of the current models is poor and further data are required to increase their robustness.

### 3.2 Predicting protection following challenge

We next determined whether protection following challenge could be predicted. A pig was considered fully protected if it had no detectable viral load (i.e. the level of virus in nasal swabs, lung and BAL was equal to zero) or no measurable pathology as assessed by one of four pathology scores (**Figure 1**). The pathology scores were gross pathology (Halbur), percent lung involvement, histopathology Morgan, and histopathology Iowa (includes nucleoprotein NP immunohistochemistry) (**Supplementary Figure S1B**).

When predicting whether a pig would be protected from challenge with H3N2 swine influenza virus following AE immunisation based on the level of N2 IgG or H3N2 IgG in serum at a single time point, there was an increase in accuracy for models based on assaying immune parameters at different sampling times after booster vaccination (D56) compared to before booster vaccination (**Figure 1**). Those models using immune parameters measured before booster vaccination were less accurate (AUC<0.8 in most cases), while those using parameters measured after booster vaccination were more accurate (AUC>0.9 in most cases). Furthermore, the models were better at predicting protection based on viral load than on pathology (**Figure 1**). When protection was predicted based on levels of pH1N1 IgG in serum, there was no clear pattern in the time points resulting in the most accurate predictions (**Figure 1**). Furthermore, predictions based on levels of pH1N1 IgG in serum were less accurate (i.e. the AUC was lower) than those based on levels of serum N2 IgG or H3N2 IgG, especially for time points after booster vaccination (**Figure 1**).

When predicting protection following IM immunisation based on the level of N2 IgG, pH1N1 IgG or H3N2 IgG in serum at a single time point, there was an increase in accuracy for models based on immune parameters assayed at time points after prime vaccination (**Figure 1**). However, models based on immune parameters assayed after booster vaccination were not necessarily more accurate than those based on immune parameters assayed between prime and booster vaccination (**Figure 1**). There was no clear indication that either levels of N2 IgG or H3N2 IgG in serum were better predictors of protection in IM immunised pigs compared with levels of pH1N1 IgG in serum. Furthermore, models based on all immune parameters were poorer at predicting protection based on gross pathology or percentage lung involvement.

For both AE and IM immunised pigs, when protection was predicted based on levels of pH1N1 or H3N2 IgA or IgG in nasal swabs, there was no clear pattern in the time points resulting in the most accurate predictions (**Supplementary Figure S5**). Furthermore, predictions based on levels of pH1N1 or H3N2 IgA or IgG in nasal swabs were less accurate than those based on levels of serum N2 IgG or H3N2 IgG. This was especially so for predictions based on levels of pH1N1 IgA, pH1N1 IgG or H3N2 IgA in nasal swabs, where AUC<0.7 in most cases (**Supplementary Figure S5**).

**Figure 2:**
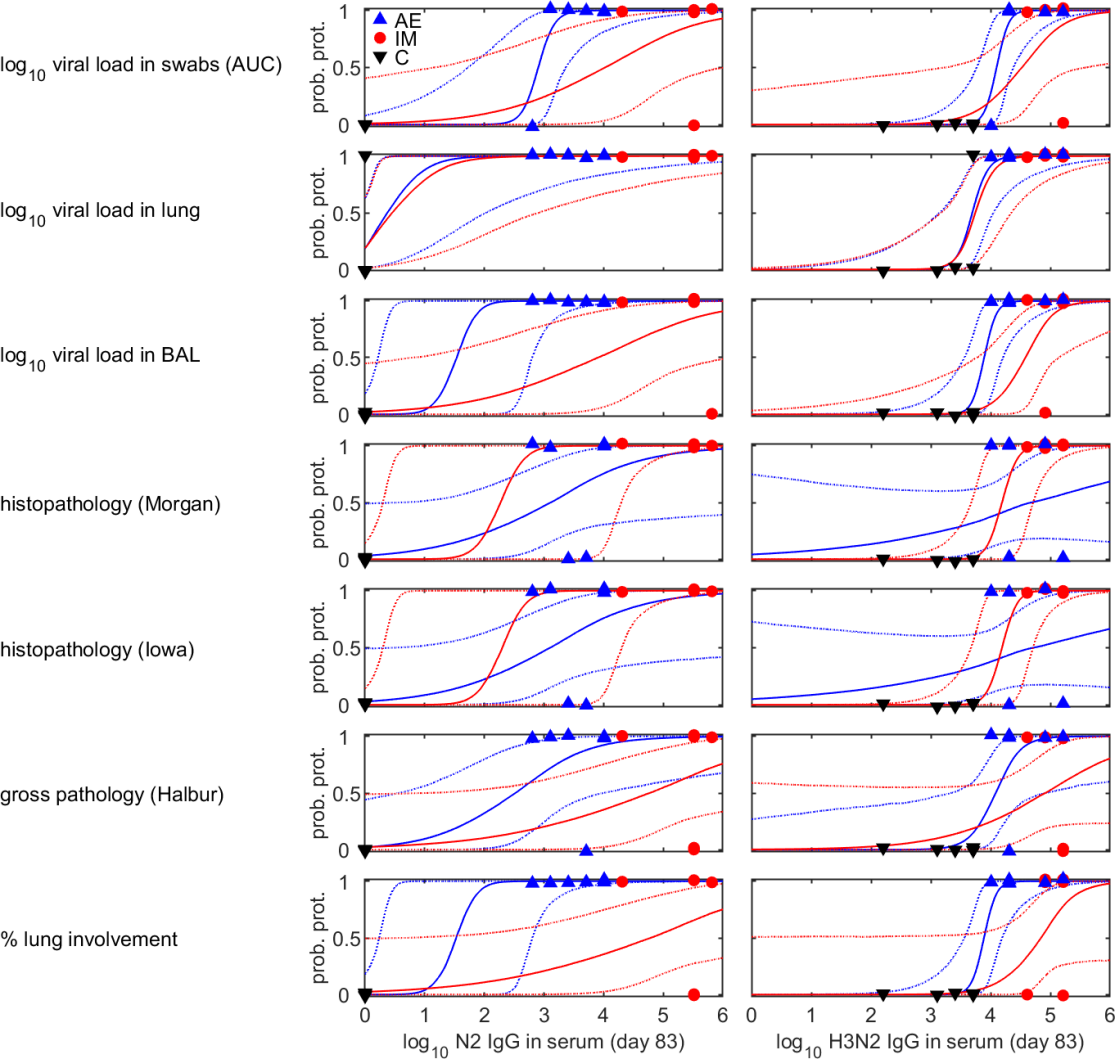
Probability of protection from viral shedding or pathology in pigs vaccinated against and challenged with H3N2 swine influenza virus and its dependence on the level of N2 IgG (left) or H3N2 IgG (right) in serum measured immediately prior to challenge (D83). Each plot shows the posterior median probability of protection (solid lines) when pigs were immunised by aerosol (blue) or intramuscularly (red), with the dotted lines showing the 95% credible intervals. The observed level of N2 or H3N2 IgG in serum for a pig and whether or not it was protected after challenge (using the measure of protection to the left of each row) are shown by the symbols: pigs immunised by aerosol (blue triangles), intramuscularly (red circles) or unvaccinated controls (black triangles).

Predictions using the level of N2 IgG or levels of H3N2 IgG in serum measured at D83 (i.e. immediately prior to challenge) all show an increasing probability of protection with increasing level of either N2 IgG or of H3N2 IgG in serum (**Figure 2**). However, the 95% credible intervals for the predicted probabilities are often wide, especially when based on the level of N2 IgG (**Figure 2**). This reflects the fact that the control pigs had no detectable N2 IgG, while all the AE and IM immunised pigs had levels >3 log_10_ (**Figure 2**).

**Figure 3:**
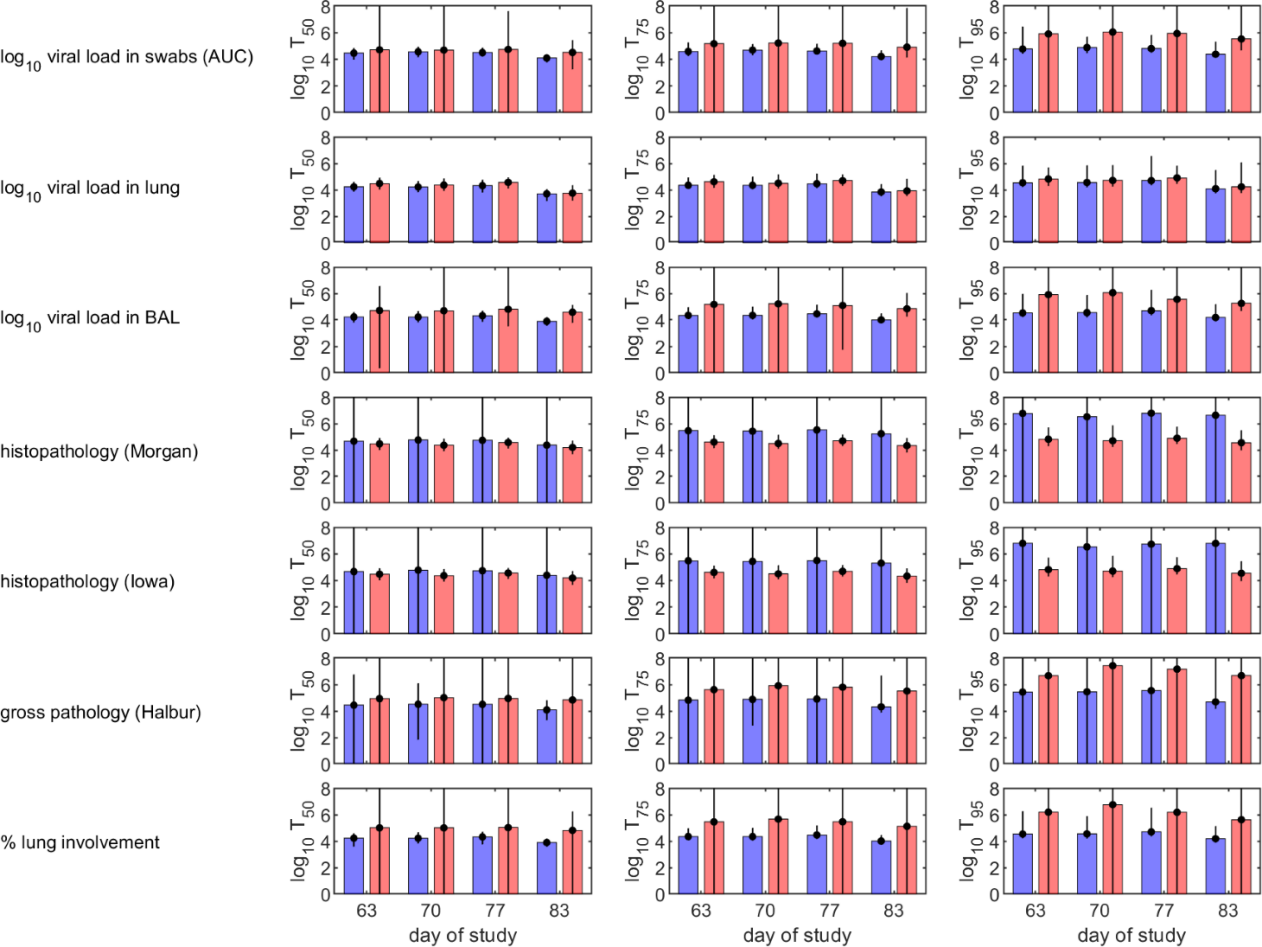
Predicted levels of H3N2 IgG in serum in vaccinated pigs required to protect 50% (left-hand column), 75% (middle column) or 95% (right-hand column) of pigs following challenge with H3N2 swine influenza virus. Pigs were immunised by aerosol (blue) or intramuscularly (red). Protection was defined as absence of virus or pathology based on different measures (listed to the left of each row). Each plot shows the posterior median (bars) and 95% credible intervals (error bars) for the required IgG levels.

The curves describing how the probability of protection changes with levels of H3N2 IgG in serum (**Figure 2**) were used to calculate the levels needed for 50%, 75% or 95% of pigs to be protected following challenge (**Figure 3**). For a given measure of protection, there was not much variation in protective titres estimated based on the level of H3N2 IgG in serum measured at different times after booster vaccination. Titres required for protection were typically higher for IM compared with AE immunised pigs, except when protection was based on histopathology. Median protective titres (log_10_ H3N2 IgG in serum) increased with the proportion of pigs protected: 3.7-4.7 (AE) and 3.7-5.0 (IM) for 50% protection; 3.8-5.5 (AE) and 3.9-5.9 (IM) for 75% protection; and 4.1-6.8 (AE) and 4.2-7.4 (IM) for 95% protection. However, there is considerable uncertainty associated with these estimates, especially for IM immunised pigs (**Figure 3**). Because of the substantial uncertainty in the estimates for the probability of protection (**Figure 2**), protective titres based on the level of N2 IgG in serum were not calculated.

In summary, these results indicate that whether or not a pig is likely to be protected from challenge can be predicted from the level of N2 IgG or the level of H3N2 IgG measured in serum shortly before challenge (i.e. within a week). Furthermore, the level of N2 IgG or H3N2 IgG in serum required for protection was lower for pigs immunised by aerosol than for those immunised intramuscularly for all measures of protection except histopathology.

## 4 Discussion

Understanding how an immune response is initiated and maintained in the lung and airways is crucial for the development of more effective vaccines against respiratory pathogens. However, measuring respiratory immune responses in humans presents significant challenges despite continuous advancement in techniques enabling direct sampling of the respiratory tract. One common approach involves obtaining of bronchoalveolar lavage (BAL), containing cells and fluid from the airways and alveoli. Based on immunization and challenge studies in pigs using viral vectored vaccines ChAdOx-NPM1-NA2 and MVA-NPM1-NA2, here we provide the first proof of principle that it is possible to predict immune parameters in BAL from immune parameters in blood (**Table 1**).

The models developed in the present study should be interpreted as demonstration that it is feasible to predict immune responses and protection, rather than as definitive models suitable for wider use. In particular, the results of model cross validation indicate that the models for immune parameters in BAL are not particularly accurate when predicting responses for pigs not included in the model fitting (**Supplementary Figure S4**), most likely reflecting problems of overfitting in the models. Predictions of whether an individual is fully protected (i.e. has no detectable virus in a particular compartment or no pathology) are perhaps more robust (**Figure 2**), but these models also require further validation. This is largely a consequence of the data used to construct the models coming from only a small number of pigs.

When developing models for the eleven immune parameters in BAL, a total of 99 potential predictors were considered (**Supplementary Table S1**), of which 30 were included in at least one model (**Table 1**). The immune parameters in blood included most frequently in the models were measurements of pH1N1 or H3N2 specific IgG. Other parameters included in multiple models include M1, NP, NA and H3N2 IFNg ELISpot responses and NA2 ELLA. The least frequently included parameters were pH1N1 and H3N2 specific IgA. Parameters measured at timepoints throughout the experiment were included in the models. For immune parameters in BAL related to H3N2 specifically, both were from timepoints after booster vaccination (D56). Overall, this suggests that future experiments should focus on measurements of pH1N1 or H3N2 specific IgG and M1, NP and H3N2 IFNg ELISpot responses after booster vaccination. These are the most informative for understanding the relationship between immune responses in blood and those in BAL. They will also help inform development of models predicting immune responses in BAL from those in blood. Initially, this should use data from studies with a similar experimental design (possibly that used here) to reduce potential confounding, but in the longer term could expand to consider data from a wider range of experiments.

Protection from influenza virus infection as determined by lack of viral shedding or lung pathology was also best predicted by responses following boost. Our results suggest that levels of either N2 or H3N2 specific IgG measured in blood within three weeks prior to challenge are suitable predictors of whether a pig is likely to be protected following challenge (**Figure 1**). Consequently, future experiments should focus on measuring these to help test and increase the robustness of the models. The models do not provide an absolute threshold level of either N2 or H3N2 specific IgG in serum above which all pigs would be protected when challenged up to three weeks later. Rather they provide a probability that a pig with a given level of IgG will be protected, which can then be used to define a threshold at which, for example, 50%, 75% or 95% of pigs would be expected to be protected (**Figure 3**). Importantly, these levels differ with route of immunisation, with lower levels required for protection in pigs immunised by aerosol compared with those immunised intramuscularly (**Figure 3**).

In this study we have used statistical modelling approaches to predict responses in BAL from those in blood and the probability of that a vaccinated animal will be protected from challenge based on IgG levels in blood. Similar approaches have been used to explore correlates of protection for influenza A in humans (28, 29). These studies estimated levels of protection based on titres measured by ELISAs for full-length haemagglutinin, haemagglutinin stalk or neuraminidase and showed increased protection with increased titres. Statistical models such as these complement mathematical models that have been used to explore viral dynamics and immune responses in human or animals hosts following influenza A infection. These models have examined many aspects of viral dynamics and the innate and adaptive immune responses to infection (30, 31). However, they have seldom been used to examine the relationship between viral titres in different compartments (e.g. in blood and in BAL or the lung) or the impact of vaccination on within-host dynamics, except for the impact of inoculum dose (32).

A recent study in young adults, who would be expected to have experienced Influenza A infections multiple times during their lives, demonstrated that intranasally delivered LAIV induced distinct, compartmentalized, antibody responses in the nasal mucosa and blood (33). The authors suggested that immunogenicity testing that relies only on peripheral blood antibody responses is likely to miss relevant mucosal antibody responses. In young children, LAIV is not only effective in preventing influenza-like illness (ILI) in the vaccinees but also resulted in a reduction in ILI in adults living in the same area, indicating reduction of viral transmission, which is not achieved after intramuscular vaccination. In contrast to the intranasal LAIV delivery, we employed aerosol administration of ChAdOx-NPM1-NA2/MVA-NPM1-NA2 ensuring broad distribution through the respiratory tract reaching deep into the lung (34). Additionally, our pigs were very recently pre-exposed to pH1N1 which may result in greater magnitude of immune responses following prime and boost immunization. We observed detectable immune responses in blood following aerosol delivery of ChAdOx-NPM1-NA2/MVA-NPM1-NA2 (15) and importantly we were able to correlate these responses with those in airways and with protection (**Figure 2**).

However, in line with the LAIV study, intranasal administration of ChAdOx-NPM1-NA2/MVA-NPM1-NA2 elicited minimal blood immune responses, providing limited protection demonstrated as reduction in viral load and lung gross pathology (15). These observations suggest that aerosol, rather than intranasal, delivery of viral vectored vaccines may offer distinct advantages. Notably millions of individuals in China have received aerosolised viral vectored SARS CoV-2 vaccines highlighting the potential feasibility of deployment at a large scale (35).

We found that that antibody responses measured after nasal swab sampling did not add to the predictive value of the models, possibly due to the variability inherent in nasal swab sampling and elution of antibody from the swabs. Sampling in humans could be greatly enhanced by using nasosorption (36) for studying mucosal antibody responses. Nasosorption has been documented to yield more concentrated antibody than alternative upper airway sampling techniques (37) and is well tolerated by human volunteers (38). Additionally, other biological fluids, such as saliva, could be explored in future studies, as IgA in sublingual and submandibular secretions has been proposed as a non-invasive marker for intestinal immune induction (39).

The next stage in developing the models for use in clinical trials will be to conduct a small clinical trial of aerosol-delivered vaccines in healthy young individuals in which BAL samples can be taken in addition to blood and nasal swabs, in order to confirm that the relationship between the different sample type, timing and assay results is maintained. A vaccine efficacy trial would then be required to test the ability of the model to predict protective responses, following which correlates of protection could be defined, obviating the need for further efficacy studies of viral-vectored influenza vaccines delivered to the lung by aerosol.

Currently licensed influenza vaccines result in low vaccine effectiveness particularly in older adults even when annual revaccination rates are high, and strategies to increase effectiveness have concentrated on higher dose or adjuvanted vaccines administered by intramuscular injection (40). Although there are obvious advantages in delivering the vaccine to the respiratory tract for improved local immunity, it has not been possible to test vaccine efficacy in humans without conducting large and expensive clinical trials, since mucosally administered vaccines result in a reduced immune response in the serum when compared to intramuscularly administered vaccines. We now present a path forward for the clinical development of influenza vaccines administered by aerosol, with the potential for reduction of the healthcare burden currently resulting from influenza each year.

## Data Availability

The original contributions presented in the study are included in the article/**Supplementary Material**. Further inquiries can be directed to the corresponding author.
