# Supplementary material for: Predicting airway immune responses and protection from immune parameters in blood following immunization in a pig influenza model
Source: Front Immunol. 2024 Dec 19;15:1506224. doi: 10.3389/fimmu.2024.1506224 (PMC11693722; doi:10.3389/fimmu.2024.1506224)
Supplement: Supplementary file 1 [file DataSheet1.pdf]

A. Experimental design prime-boost immunization experiment

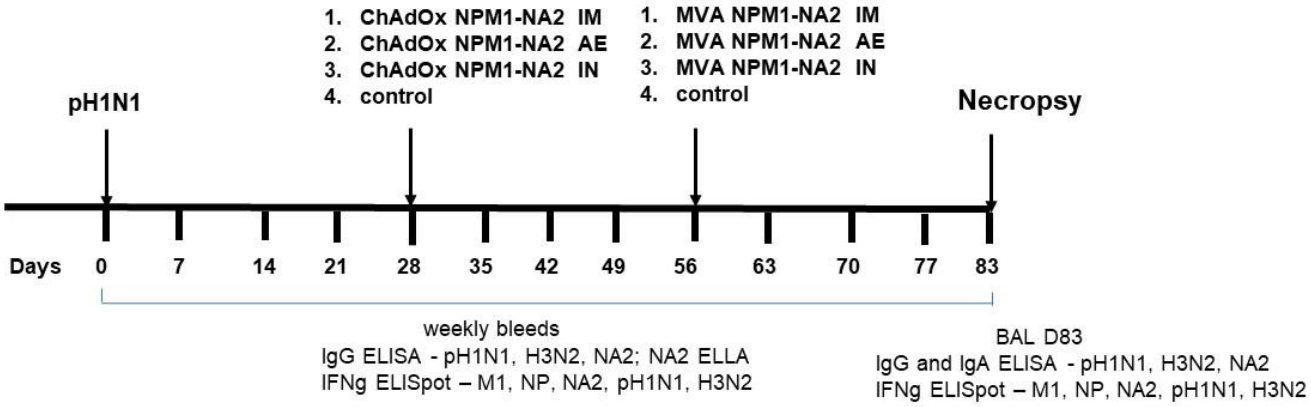

B. Experimental design H3N2 challenge experiment

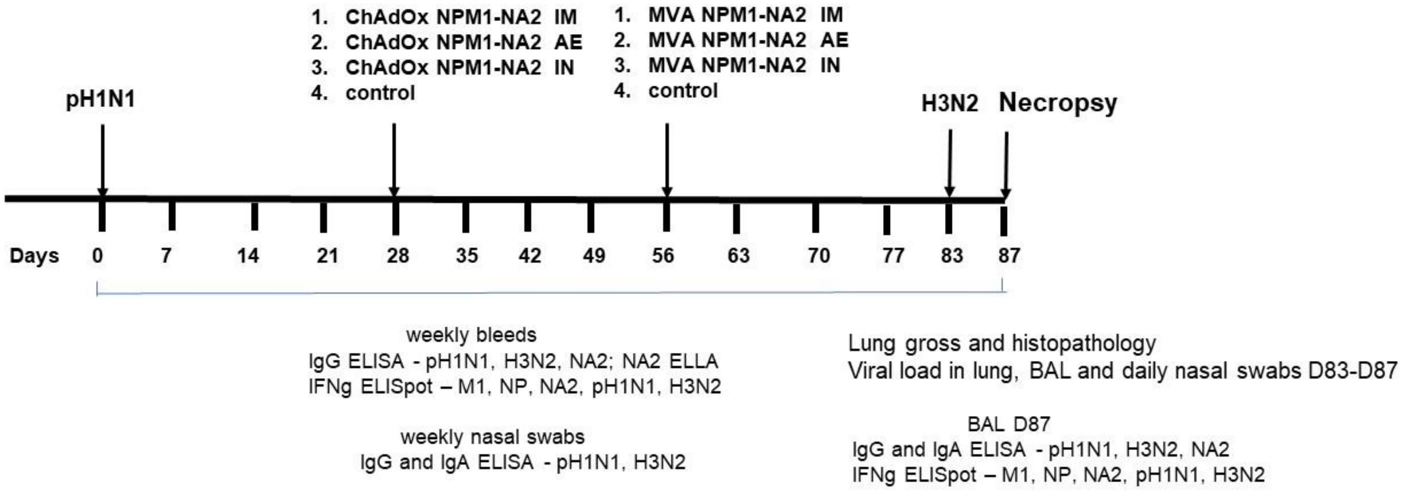

Supplementary Figure 1. Experimental design of pig studies. A) Twenty four pigs were inoculated with pH1N1. Four weeks later the pigs were randomly divided into four groups of five animals and were immunized with ChAdOx2-NPM1-NA2 intramuscularly (IM), intranasally (IN) or by aerosol (AE). Four weeks after the ChAdOx2-NPM1-NA2 immunization the pigs were boosted by the same delivery route with MVA-NPM1-NA2. Unimmunized, but pH1N1 pre-exposed pigs were used as controls (C). The animals were culled four weeks after the boost, and immune responses were evaluated in the bronchoalveolar lavage (BAL), spleen and blood at the indicated time points. **B)** Twenty-four animals were inoculated with pH1N1. Four weeks later the pigs were randomly divided into four groups of six animals and immunized either IM, IN or by AE with ChAdOx2-NPM1-NA2 and MVA-NPM1-NA2 four weeks apart. Four weeks after the MVA-NPM1-NA2 boost, all animals were infected intranasally with H3N2 virus**.** Animals were culled four days later and immune responses, viral load and lung pathology assayed at the indicated tissues and time points.

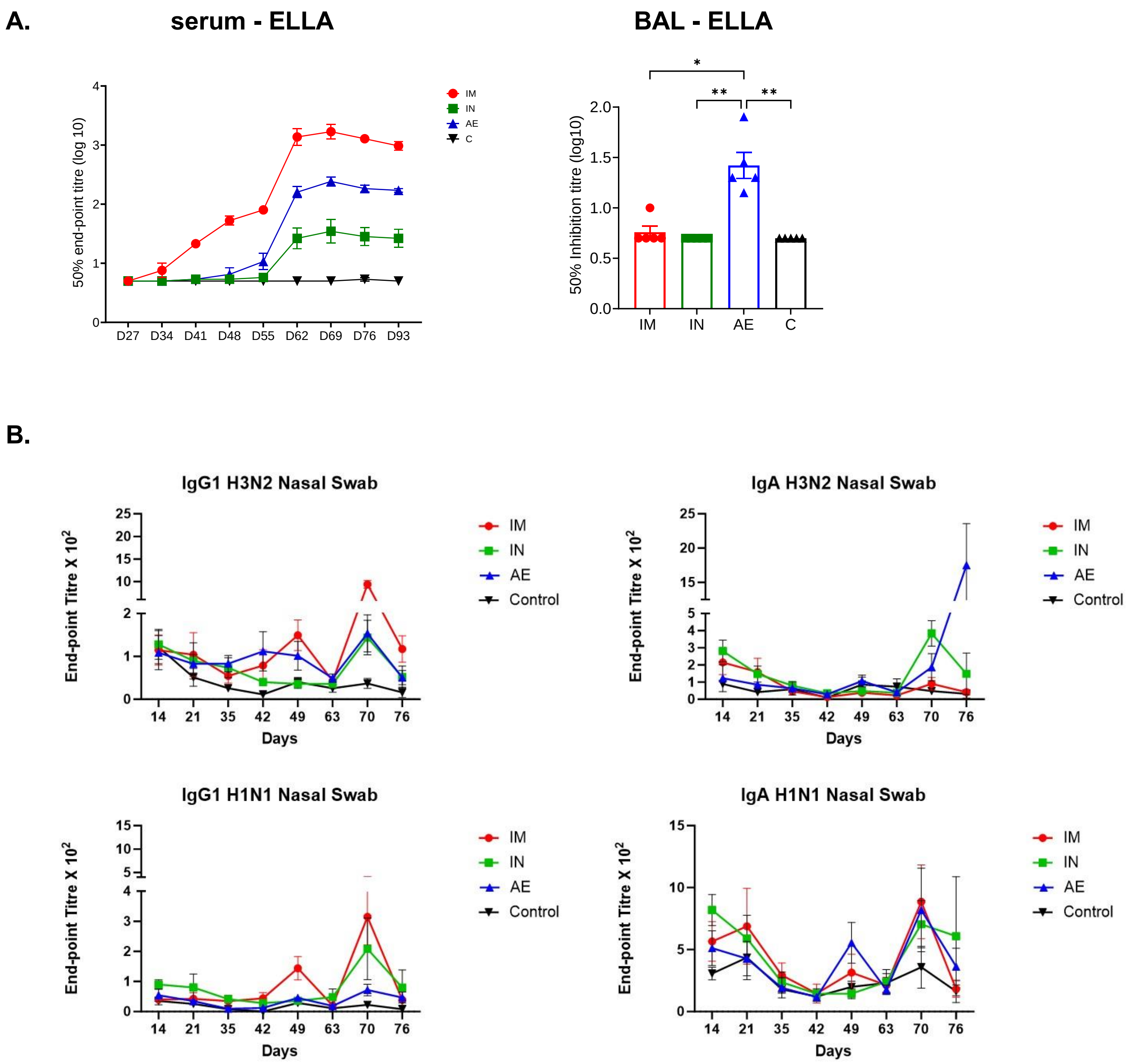

Supplementary Figure 2. Neuraminidase inhibition antibody titres in serum and BAL and ELISA titres in nasal swabs. A) Enzyme-Linked Lectin Neuraminidase Inhibitory Antibodies Assay (ELLA) was performed on serum and BAL samples from the pre-exposure immunization study. **B)** pH1N1 and H3N2 specific IgG and IgA responses in nasal swabs in the challenge study were determined by ELISA at the indicated time points. The mean and standard error (SEM) is presented in each time point. Asterisks denote significance between indicated groups (*p<0.05, **p<0.01).

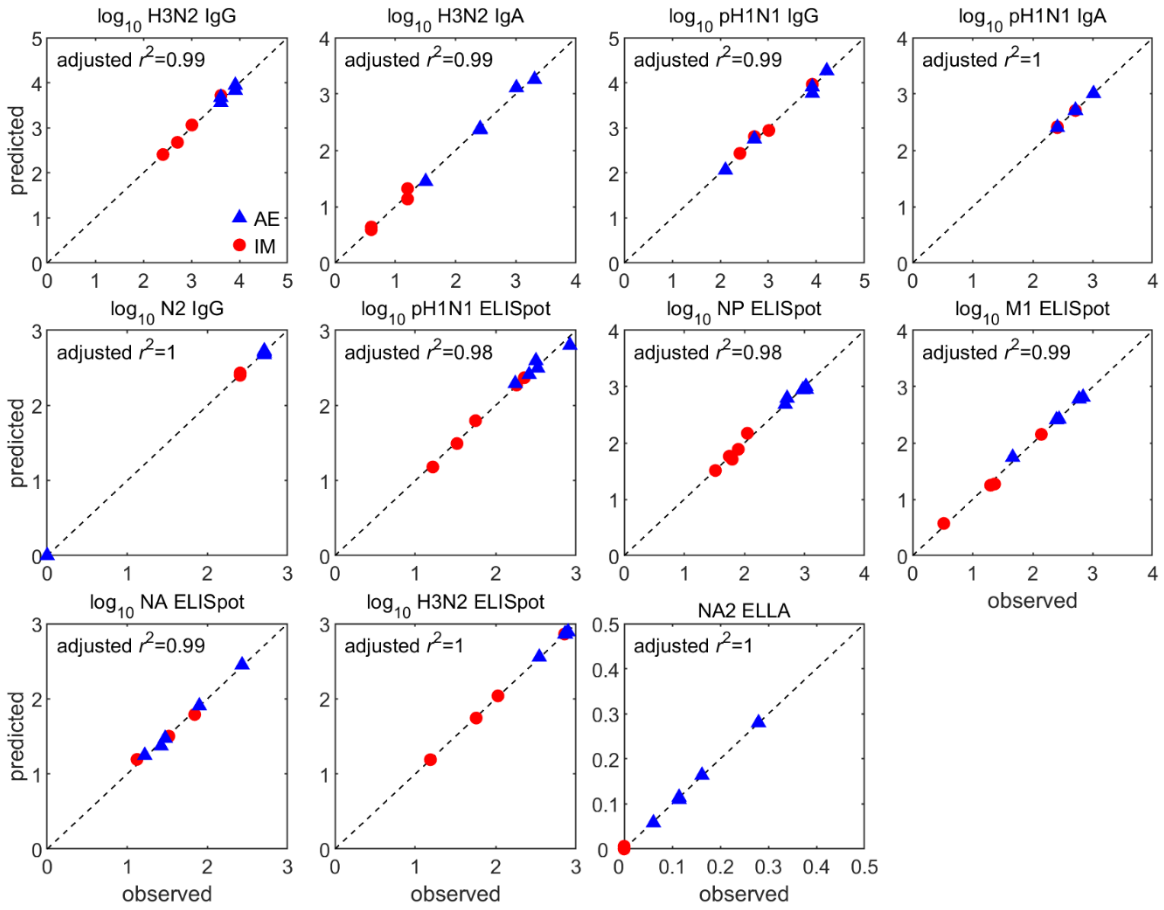

Supplementary Figure 3. Assessment of models predicting immune responses against influenza virus in pigs in broncho-alveolar lavage (BAL) from those measured in blood. Each plot shows the observed level of the measure in BAL (indicated above the plot) plotted against the level predicted by the model. Pigs were immunised by aerosol (blue triangles) or intramuscularly (red circles). The black dashed lines indicates where the observed equals the predicted values.

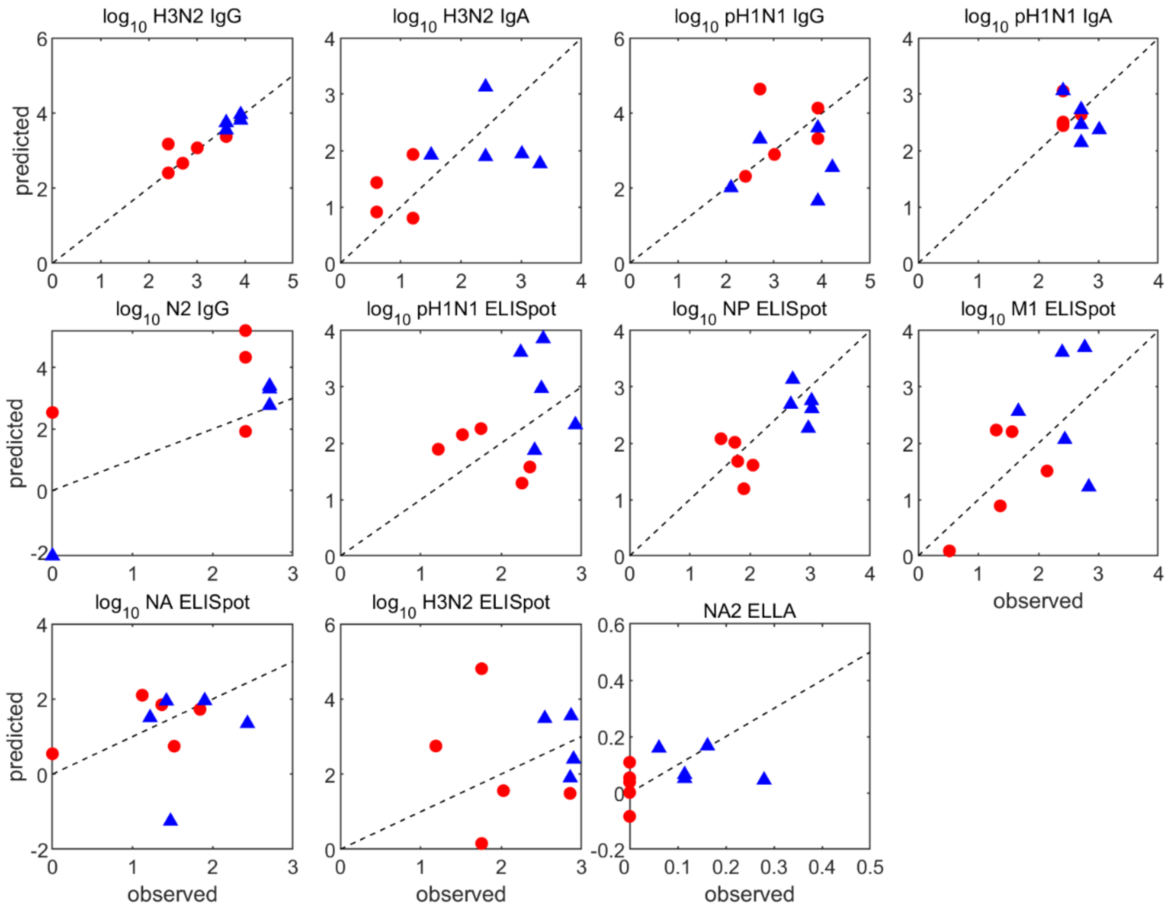

Supplementary Figure 4. Leave-one-out cross validation of models predicting immune responses against influenza virus in pigs in broncho-alveolar lavage (BAL) from those measured in blood. Each plot shows the observed level of the measure in BAL (indicated above the plot) in a pig plotted against the level predicted by a model fitted to a dataset omitting the data for the pig. Pigs were immunised by aerosol (blue triangles) or intramuscularly (red circles). The black dashed lines indicates where the observed value equals the predicted value.

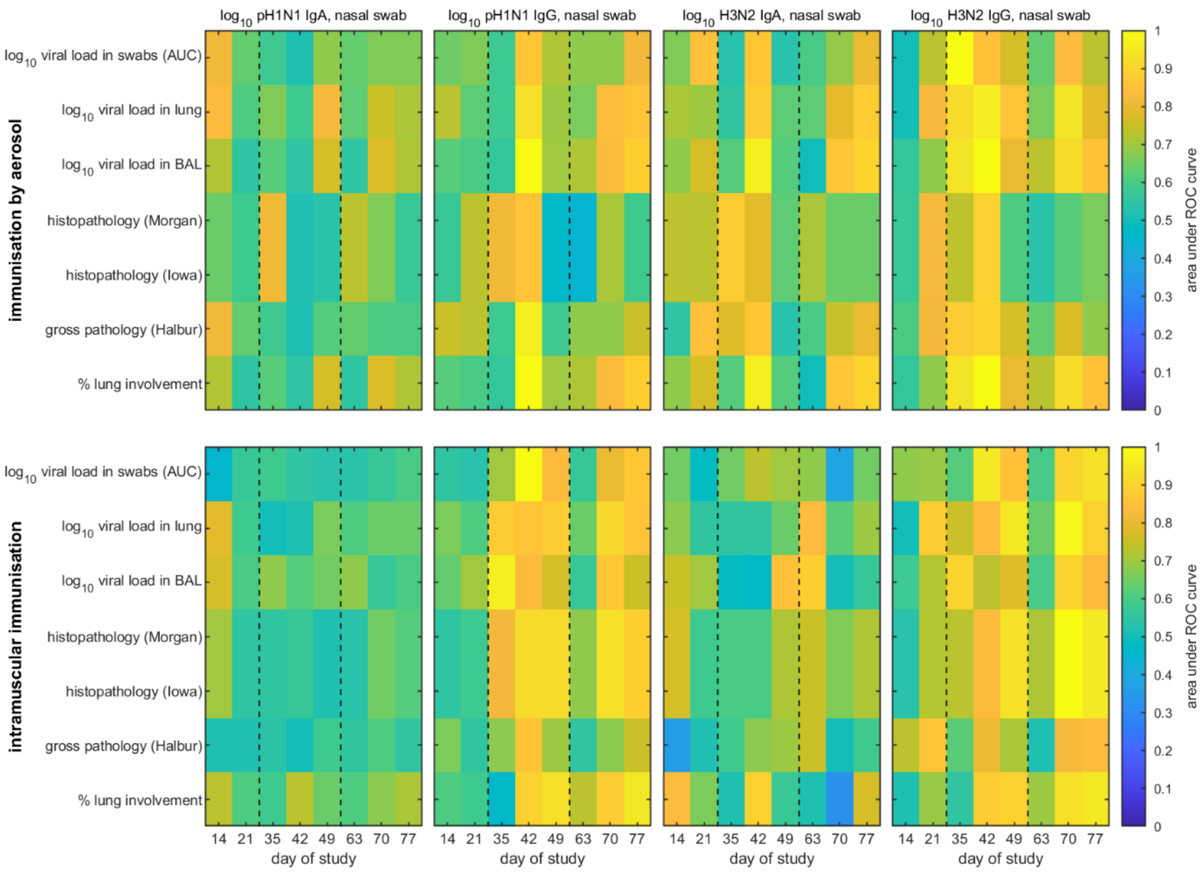

Supplementary Figure 5. Receiver operator characteristic (ROC) analysis of models for the probability of protection in pigs following challenge with H3N2 swine influenza virus. Pigs were immunised by aerosol (top) or intramuscularly (bottom). Protection was defined as absence of virus or pathology based on different measures (listed on the *y* axis), while the probability of protection was estimated using each immune parameter (indicated above the panel) measured at a single time point (listed on the *x* axis) as a predictor. The black dashed lines indicate the timing of prime and boost vaccinations.

Supplementary Table 1. Immune parameters in blood used as predictors in models for immune parameters against influenza virus in pigs in broncho-alveolar lavage (BAL).

immune parameter	time points (study day)
log_{10} H3N2 IgG in serum	7, 14, 21, 28, 35, 42, 49, 56, 62, 69, 76, 83
log_{10} pH1N1 IgG in serum	7, 14, 21, 28, 35, 42, 49, 56, 62, 69, 76, 83
log_{10} N2 IgG in serum	28, 35, 42, 49, 55, 62, 69, 76, 83
log_{10} H3N2 IgA in serum	28, 35, 42, 56, 63
log_{10} pH1N1 IgA in serum	27, 34, 41, 55, 62, 69, 83
log_{10} pH1N1 ELISpot (PBMC*)	28, 35, 42, 49, 56, 63, 70, 77, 83
log_{10} NP ELISpot (PBMC)	28, 35, 42, 49, 56, 63, 70, 77, 83
log_{10} M1 ELISpot (PBMC)	28, 35, 42, 49, 56, 63, 70, 77, 83
log_{10} NA ELISpot (PBMC)	28, 35, 42, 49, 56, 63, 70, 77, 83
log_{10} H3N2 ELISpot (PBMC)	28, 35, 42, 49, 56, 63, 70, 77, 83
NA2 ELLA (serum)	27, 34, 41, 55, 62, 69, 76, 83

* PBMC: peripheral blood mononuclear cells
